# Supplementary material for: Dynalign II: common secondary structure prediction for RNA homologs with domain insertions
Source: Nucleic Acids Res. 2014 Nov 21;42(22):13939–48. doi: 10.1093/nar/gku1172 (PMC4267632; doi:10.1093/nar/gku1172)
Supplement: SUPPLEMENTARY DATA [file supp_gku1172_nar-02021-z-2014-File012.zip › manual/javadoc/allclasses-frame.html]

All Classes


# All Classes

- AnnotationMenu
- ApplicationRootFrame
- BorderBuilder
- BorderedButtonPanel
- ConstraintsMenu
- DotPlotBackend
- DotPlotBackendProxyJNI
- DotsMenu
- DrawingWindow
- DynalignFoldWindow
- DynalignRefoldWindow
- DynamicMenuBar
- Efn2Window
- FieldPanel
- FieldPanel.FilePanel
- FieldPanel.NumberPanel
- FileChooser
- FileChooser.Types
- FileField
- FileMenu
- FoldDoubleWindow
- FoldSingleWindow
- FoldSuboptimalWindow
- HelpMenu
- HTMLCheckBox
- ImageDialog
- ImageGrabber
- InternalWindow
- MacChecker
- MaxExpectWindow
- ModuleWindow
- MultilignWindow
- MultiWindow
- NucleicAcidMenu
- NumberField
- NumberField.DoubleField
- NumberField.FloatField
- NumberField.IntegerField
- OligoScreenWindow
- OligoWalkWindow
- PartitionDoubleWindow
- PartitionSingleWindow
- PlotDialog
- PlotMenu
- PlotRunner
- ProbKnotWindow
- PseudoknotWindow
- RadioButtonPanel
- RefoldWindow
- RNAstructure
- RNAstructureBackendCalculator
- RNAstructureBackendCalculatorProxyJNI
- RNAstructureMenu
- RolloverMenu
- RunnerTemplate
- ScrollerPane
- SequenceDisplayWindow
- SimpleDialogHandler
- StochasticWindow
- StructureBackend
- StructureBackendProxyJNI
- StructureDialog
- StructureMenu
- StructureRunner
- TurboFoldWindow
- ValueSelectionDialog
